# Spray Application of Nonpathogenic Fusaria onto Rice Flowers Controls Bakanae Disease (Caused by Fusarium fujikuroi) in the Next Plant Generation

**DOI:** 10.1128/AEM.01959-20

**Published:** 2021-01-04

**Authors:** Hiroki Saito, Mai Sasaki, Yoko Nonaka, Jun Tanaka, Tomomi Tokunaga, Akihiro Kato, Tran Thi Thu Thuy, Le Van Vang, Le Minh Tuong, Seiji Kanematsu, Tomotaka Suzuki, Kenichi Kurauchi, Naoko Fujita, Tohru Teraoka, Ken Komatsu, Tsutomu Arie

**Affiliations:** a Laboratory of Plant Pathology, Graduate School of Agriculture, Tokyo University of Agriculture and Technology (TUAT), Fuchu, Tokyo, Japan; b United Graduate School of Agriculture, TUAT, Fuchu, Tokyo, Japan; c Department of Plant Protection, College of Agriculture and Applied Biology, Can Tho University, Can Tho City, Vietnam; d Tohoku Agricultural Research Center, NARO, Morioka, Iwate, Japan; e Miyagi Furukawa Agricultural Experiment Station, Osaki, Miyagi, Japan; f Aomori Prefectural Industrial Technology Research Center, Kuroishi, Aomori, Japan; g Institute of Global Innovation Research (GIR), TUAT, Fuchu, Tokyo, Japan; Nanjing Agricultural University

**Keywords:** bakanae disease, biocontrol, flower spraying, *Fusarium fujikuroi*, nonpathogenic *Fusarium commune*, seed-borne

## Abstract

Bakanae disease, caused by Fusarium fujikuroi, is an economically important seed-borne disease of rice. F. fujikuroi is horizontally transmitted to rice flowers and vertically transmitted to the next generation via seeds. The fungus induces typical symptoms such as abnormal tissue elongation and etiolation. Sanitation of seed farms and seed disinfection are the only effective means to control bakanae disease at present; however, the efficacy of these methods is often insufficient. Therefore, alternative and innovative control methods are necessary. We developed a novel method for applying nonpathogenic fusaria as biocontrol agents by spraying spore suspensions onto rice flowers to reduce the incidence of seed-borne bakanae. We visualized the interaction between Fusarium commune W5, a nonpathogenic fusarium, and Fusarium fujikuroi using transformants expressing two different fluorescent proteins on/in rice plants. W5 inhibited hyphal extension of F. fujikuroi on/in rice flowers and seedlings, possibly by competing with the pathogen, and survived on/in rice seeds for at least 6 months.

**IMPORTANCE** We demonstrated that a spray treatment of rice flowers with the spores of nonpathogenic fusaria mimicked the disease cycle of the seed-borne bakanae pathogen Fusarium fujikuroi and effectively suppressed the disease. Spray treatment of nonpathogenic fusaria reduced the degree of pathogen invasion of rice flowers and vertical transmission of the pathogen to the next plant generation via seeds, thereby controlling the bakanae disease. The most promising isolate, F. commune W5, colonized seeds and seedlings via treated flowers and successfully inhibited pathogen invasion, suggesting that competition with the pathogen was the mode of action. Seed-borne diseases are often controlled by seed treatment with chemical fungicides. Establishing an alternative method is a pressing issue from the perspectives of limiting fungicide resistance and increasing food security. This work provides a potential solution to these issues using a novel application technique to treat rice flowers with biocontrol agents.

## Abstract (precis)

We demonstrated that a spray treatment of rice flowers with the spores of nonpathogenic fusaria mimicked the disease cycle of the seed-borne bakanae pathogen Fusarium fujikuroi and effectively suppressed the disease. Spray treatment of nonpathogenic fusaria reduced the degree of pathogen invasion of rice flowers and vertical transmission of the pathogen to the next plant generation via seeds, thereby controlling the bakanae disease. The most promising isolate, F. commune W5, colonized seeds and seedlings via treated flowers and successfully inhibited pathogen invasion, suggesting that competition with the pathogen was the mode of action. Seed-borne diseases are often controlled by seed treatment with chemical fungicides. Establishing an alternative method is a pressing issue from the perspectives of limiting fungicide resistance and increasing food security. This work provides a potential solution to these issues using a novel application technique to treat rice flowers with biocontrol agents.

## INTRODUCTION

Bakanae, or foolish seedling, caused by Fusarium fujikuroi Nirenberg (also known as Gibberella fujikuroi Sawada mating population C [MP-C]), is a highly destructive seed-borne rice (Oryza sativa L.) disease that is distributed throughout almost all of the rice-growing areas of the world (1). This disease was first described by Hori (2) in Japan. Most strains of this pathogen induce typical bakanae symptoms such as etiolation and abnormal elongation of the leaves in seedlings and mature plants resulting from the production of gibberellic acid (3). F. fujikuroi is also known as a producer of fusaric acid, beauvericin, and other secondary metabolites (4). There are also some reports of a few strains of F. fujikuroi that produce fumonisins B1, B2, and B3 instead of gibberellic acid; however, the fumonisins do not cause elongation but induce stunting and early withering (5, 6).

F. fujikuroi invades rice plant tissues, and, according to the report by Nisikado and Kimura (7), microconidia and mycelia especially accumulate in pitted vessels but not in the phloem or in parenchyma cells. The pathogen conidiates on the basal sheath joint of rice plants, and conidia or sometimes ascospores are dispatched and horizontally transmitted, often by wind, to the flowers of surrounding rice plants during the flowering period (Fig. S1 in the supplemental material) (8, 9). The F. fujikuroi-infected flowers produce infested seeds, and the pathogen is vertically transmitted to the next plant generation (8). In most cases, seeds infested with the pathogen show no visible symptoms, hindering the identification and elimination of bakanae-infested seeds among all harvested seeds (10). Horizontal transmission of this pathogen also occurs in seed-soaking containers that are used to hasten germination (11) and also in nursery trays used to raise seedlings (12). Some of the infested seedlings show no or indistinct symptoms that tend to be overlooked by farmers; thus, some infested seedlings are transplanted into paddy fields and will serve as sources of inocula (Fig. S1) (3).

At present, seed-borne diseases, including bakanae disease, are effectively controlled by the application of chemical fungicides (e.g., ipconazole) onto rice seeds (13). However, such application often leads to the emergence of fungicide-resistant strains of the pathogens, an issue of great concern. Indeed, due to the repeated application of benzimidazole fungicides (e.g., benomyl) to control seed-borne diseases, benzimidazole-resistant pathogens frequently emerged in the 1970s worldwide (14–16), followed by the discovery of benzimidazole-resistant F. fujikuroi in Japan in 1980 (17). Now, at last, benomyl is no longer recommended for use as a bakanae-control fungicide. Therefore, although ipconazole can effectively control bakanae disease at present, in consideration of the possible future emergence of resistant strains and a trend of reducing the use of chemical fungicides, alternative methods are needed.

In the case of suppressing bakanae disease without chemicals, biological control using the following bacterial or fungal agents has been studied: Fusarium oxysporum, *Trichoderma* spp. (18–20), *Talaromyces* spp. (21, 22), Metschnikowia pulcherrima, Pichia guilliermondii, Sporidiobolus pararoseus (23), Pseudomonas fluorescens, and *Bacillus* spp. (24–26). Some of the fungal agents listed above, such as Trichoderma asperellum SKT-1 and Talaromyces flavus SAY-Y-94-01, have already been put into practical use in seed and nursery tray treatments. It is also becoming common to disinfect seeds using hot water in Japan; this technique has been demonstrated at the laboratory scale (60°C, 10 min) (27) and for practical use (62.7 to 64.8°C, 5 to 15 min) (28). Hot water disinfection is also effective against other seed-borne pathogens such as Pyricularia oryzae and Burkholderia plantarii (29).

The effects of these alternative methods are still somewhat unstable and lead to outbreaks of bakanae disease (30). Therefore, we need to develop an innovative method to overcome these issues to stably control bakanae disease.

In this study, we hypothesized that treating flowers with biocontrol agents would imitate the disease cycle of the bakanae pathogen (Fig. S1) and could be a solution for controlling the disease. Therefore, we focused on using nonpathogenic *Fusarium* spp. (NPFs) that are frequently isolated from rice plant tissues (31) to develop a method for using them as biocontrol agents for bakanae disease.

(Portions of this research have been published as abstracts from oral presentations [32, 33].)

## RESULTS

### Selection of biocontrol agents against bakanae disease.

**FIG 1:**
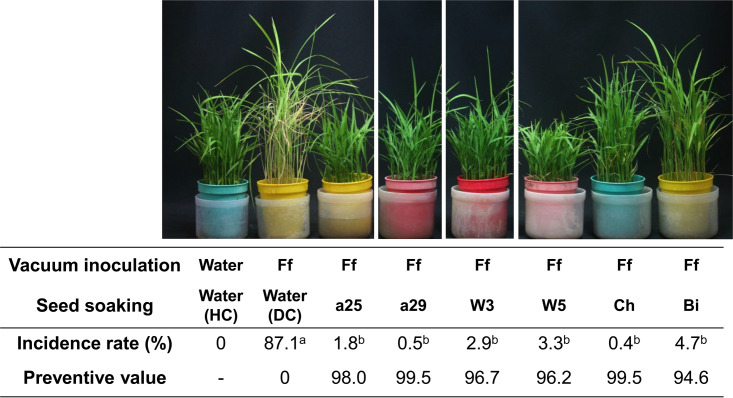
Biocontrol efficacy of seed treatments with selected nonpathogenic *Fusarium* isolates on bakanae disease in rice (Oryza sativa) cv. Tanginbouzu. Artificially infested seeds were prepared by vacuum inoculation in bud-cell suspensions of F. fujikuroi Miyagi 92-10 (Ff). Infested seeds were soaked in each of the following suspensions: bud-cell suspensions of selected nonpathogenic *Fusarium* isolates (a25, a29, W3, or W5), ipconazole (Ch), conidial suspension of Trichoderma asperellum SKT-1 (Bi), or water (DC, diseased control). Noninfested seeds without any treatment were used as healthy controls (HC). The incidence rate was calculated as a percentage of the diseased plant number per total plant number for each treatment at 14 days after sowing. The preventive value was calculated with the following formula: (preventive value) = [(incidence rate of DC) – (incidence rate for each treatment)] × 100/(incidence rate of DC). In the “Incidence rate (%)” line, different letters indicate significant differences between the treatments by Tukey’s test (*P < *0.05).

We obtained 106 *Fusarium* isolates from rice plant tissues and initially selected eight isolates that had a lower than 33.4% incidence rate in at least one ice cube tray test trial (Table S1 in the supplemental material). The eight isolates were subjected to a secondary screening in pots to finally select the following four *Fusarium* isolates: W3 (isolated from rice tissues from Osato, Miyagi, Japan, in 2011), W5 (isolated from Inakadate, Aomori, Japan, in 2011), a25, and a29 (both isolated from Tien Giang, Vietnam, in 2008). All four selected isolates repeatedly and efficiently controlled bakanae disease in which seeds were treated with a bud-cell suspension in pot tests (Fig. 1). The bakanae prevention values for these four strains were higher than 95, a value that did not differ significantly from those of biological fungicide (Bi) Eco-hope (T. asperellum SKT-1) and chemical fungicide (Ch) Techlead-C Flowable (ipconazole). This result indicated that these isolates suppressed at least horizontal transmission of the pathogen between seedlings, a frequent occurrence in nursery trays and the common target for control.

Based on morphological and molecular characteristics (ribosomal DNA [rDNA] internal transcribed spacer [ITS] and intergenic spacer [IGS] sequences), we identified W3 (GenBank accession nos. LC516588 and LC516583) and W5 (GenBank accession nos. LC516587 and LC516582) as Fusarium commune and a25 (GenBank accession nos. LC516589 and LC516584) and a29 (GenBank accession nos. LC516590 and LC516585) as Fusarium proliferatum (Fig. S2). The molecular phylogenetic tree using the IGS region (Fig. S2B) indicates that these four fusaria are distant relatives of Fo47, a well-known nonpathogenic F. oxysporum strain used as a biocontrol agent of soilborne diseases. Some isolates of F. commune are pathogenic to some plant species, including tomato (34), maize (35), and conifer seedlings (36, 37), and some isolates of F. proliferatum are pathogenic to garlic (38, 39), onion (39), blueberry (40), and other crops. The four selected isolates, however, did not show any significant symptoms on tomato (Solanum lycopersicum), cabbage (Brassica oleracea), or rice when inoculated by a standard drench method or by a spray method (Fig. S3). Thus, we consider the four isolates to be nonpathogenic fusaria that can be promising biocontrol agents.

### Control of bakanae disease by spraying rice flowers with nonpathogenic *Fusarium* isolates.

Considering the life cycle of F. fujikuroi, which infects rice flowers and is transmitted to the next plant generation via seeds (Fig. S1), we hypothesized that the nonpathogenic fusaria we selected could also be transmitted via seeds and might suppress bakanae disease in plants of the next generation.

**FIG 2:**
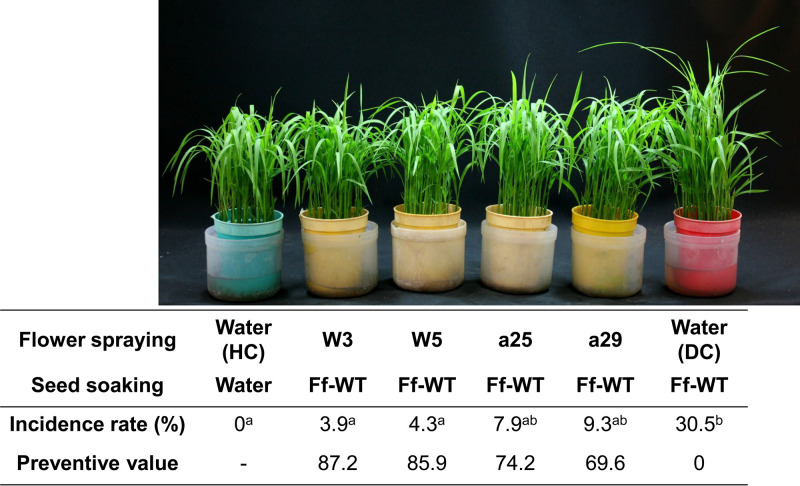
Biocontrol efficacy of spraying flowers with selected nonpathogenic *Fusarium* spp. on bakanae disease in rice (Oryza sativa) cv. Tanginbouzu. Flowers of healthy rice plants were sprayed with bud-cell suspensions of selected nonpathogenic *Fusarium* isolates (a25, a29, W3, or W5) or water (HC or DC). Harvested seeds were soaked in bud-cell suspensions of F. fujikuroi Miyagi 92-10 (Ff) or water as the control (HC). The incidence rate and preventive values were calculated as described in the legend to Fig. 1. Different letters indicate significant differences between the treatments by Tukey’s test (*P* < 0.05).

To test this hypothesis, we harvested seeds from rice plants whose flowers had been sprayed with a bud-cell suspension of W3, W5, a25, or a29 in a growth chamber that was free from the bakanae pathogen. The seeds were then inoculated using the bud-cell suspension of F. fujikuroi and were sown on sterilized soil in a pot. Similar to the direct treatment of seeds with the nonpathogenic fusaria, seeds generated from the spray-treated flowers had a significantly lower occurrence of bakanae disease (Fig. 2) than flowers sprayed with water (control). This result suggested that the four isolates successfully colonized seeds after flower treatments and were vertically transmitted to the next generation of plants. Moreover, spraying flowers with a biocontrol agent and using the seeds generated from the sprayed flowers can be a novel method for controlling the emergence of bakanae disease in nursery trays.

### Spraying biocontrol agents onto rice flowers in paddy fields.

We hypothesized that spray treatment of the biocontrol agents onto flowers would suppress the bakanae pathogen infection and reduce the vertical transmission of the pathogen to the next plant generation via seeds. Therefore, we reproduced the horizontal transmission conditions of the bakanae pathogen in paddy fields and tested whether spray treatment of the biocontrol agents onto flowers could suppress the horizontal transmission of the bakanae pathogen to flowers and its subsequent vertical transmission via seeds.

Flowers of rice cv. Tanginbouzu were sprayed with bud-cell suspensions of the nonpathogenic fusaria (and T. asperellum as a control in 2013 and 2014). For the inocula, F. fujikuroi-infested rice cv. Koshihikari were planted in a paddy field as shown in the schematic experimental design (Fig. S4). In this field test, we tested only F. commune W3 and W5 because F. proliferatum a25 or a29 isolated from Vietnamese rice are regulated by the Japanese Plant Quarantine Law, and, moreover, F. proliferatum is known as a possible producer of fumonisin, a mycotoxin (41).

Spraying W3 or W5 onto rice flowers in the paddy field significantly reduced the occurrence of bakanae disease in the next generation of plants in comparison with the control water treatment (Table 1). Additionally, seed quality, including 1,000-grain weight, germination rate, and appearance, was not significantly affected by treating flowers with W3 or W5 (Table 1). Note that seed quality was not affected even in the control (DW; sprayed with distilled water) treatment, a finding that is consistent with the fact that in practical rice production, latent contamination of bakanae-infested seeds among seed stocks is a problem. Under the conditions of our experiments, spraying flowers with W3 and W5 did not negatively affect rice seed quality.

**TABLE 1:** Effect of spraying rice (Oryza sativa) flowers with nonpathogenic Fusarium commune on seed quality and the appearance of bakanae disease^*k*^

Yr	Treatment	Seed quality			Biocontrol effect	
		GV^*a*^ (%)	GP^*b*^ (%)	GW^*c*^ (g)	IR^*d*^ (%)	PV^*e*^
2012	DW^*f*^	NT^*j*^	96.25a	24.90a	14.4a	0.0
	W3^*g*^	NT	98.75a	25.26a	5.6ab	60.9
	W5^*h*^	NT	98.75a	25.46a	1.3b	91.2
	*Trichoderma*^*i*^	NT	NT	NT	NT	NT
2013	DW	NT	97.20a	25.59a	24.5a	0.0
	W3	NT	96.75a	26.02a	11.0b	54.9
	W5	NT	97.00a	25.78a	8.2b	66.4
	*Trichoderma*	NT	98.25a	25.59a	7.0b	71.4
2014	DW	97.20a	97.75a	26.64a	5.5ab	0.0
	W3	96.75a	97.00a	26.82a	2.6b	53.5
	W5	97.00a	98.50a	26.56ab	1.4b	75.2
	*Trichoderma*	98.25a	99.25a	26.28b	8.9a	−60.4
2015	DW	91.75a	93.00a	25.63a	14.0a	0.0
	W3	NT	NT	NT	NT	NT
	W5	88a	91.75a	25.33a	2.6b	81.2
	*Trichoderma*	NT	NT	NT	NT	NT

a GV, germination vigor of 100 seeds.

b GP, germination percentage of 100 seeds.

c GW, one-thousand-grain weight.

d IR, incidence rate (diseased plant number × 100/total plant number).

e PV, preventive value [(IR of DW − IR of each treatment) × 100/IR of DW].

f DW, spraying sterile distilled water (control).

g Spraying bud cell suspension of Fusarium commune W3.

h Spraying bud cell suspension of F. commune W5.

i Spraying conidial suspension of Trichoderma asperellum isolated from commercial biofungicide.

j NT, not tested.

k Every value indicates the mean value for four field plot replicates. Different letters in the same column indicate significant differences between the treatments by Tukey’s test (*P < *0.05). In each year, every evaluation was repeated three times, and the mean value for four experimental plots with the same treatment was obtained for each evaluation item.

These results indicated that spray treatments of W3 or W5 onto rice flowers had a robust effect on controlling bakanae disease in a field environment and could be used practically in paddy fields. Moreover, we recommend that the application of biocontrol agents onto flowers should be performed only in fields for seed production. Such an effort is not necessary for rice grain production; thus, it is possible to refrain from using the biocontrol agents beyond necessity. In comparison to the nonpathogenic F. commune W3 and W5, the preventive value in T. asperellum was not stable (Table 1), suggesting that the efficacy of spray treatments onto rice flowers may depend on the agent. In addition, there were no differences among rice cultivars in terms of the biocontrol efficacy of W3 or W5 (Fig. S5).

### Survivability of W5 on/in the seeds and seedlings generated from rice flowers sprayed with W5.

We have proposed that biocontrol agents sprayed onto flowers could latently colonize the seeds produced by the flowers and could be transmitted vertically to the next generation of plants. In order to support this hypothesis, we tracked the colonization of W5 on/in the rice seeds that developed from flowers after spray treatments with W5.

To visualize W5 spores and mycelia, we generated a W5 transformant, designated W5-GFP-GEN^r^, that expressed enhanced green fluorescent protein (EGFP) and Geneticin resistance. W5-GFP-GEN^r^ constantly produced a green fluorescent signal in mycelia, microconidia, macroconidia, chlamydospores, and bud cells (Fig. S6). We also confirmed that the biocontrol efficacy of W5-GFP-GEN^r^ against bakanae disease was equivalent to that of the wild-type W5 (Fig. S7). These results prompted us to analyze the dynamics of the biocontrol agent *in planta* and the interactions between the pathogen and the biocontrol agent using W5-GFP-GEN^r^.

**FIG 3:**
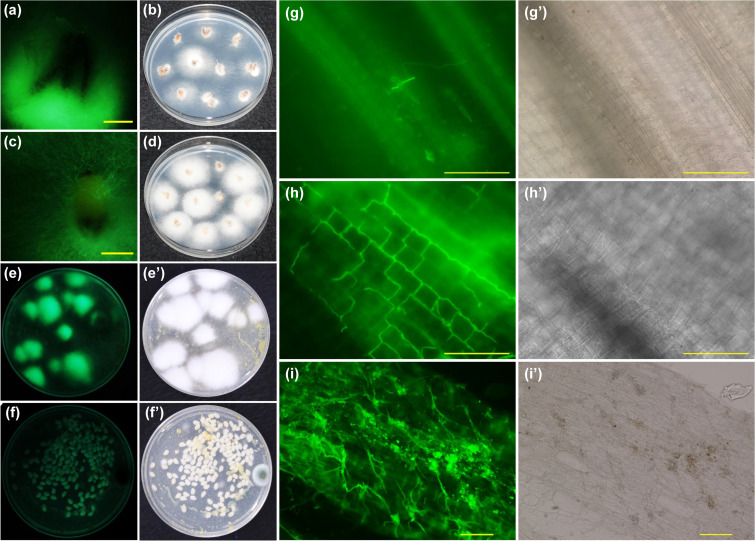
Reisolation and observation of nonpathogenic Fusarium commune W5-GFP-GEN^r^ on rice (Oryza sativa) seeds and seedlings from the next plant generation produced from flowers sprayed with W5-GFP-GEN^r^. Seeds stored for 6 months were separated into rice hulls and husked rice; husked rice was separated into bran and polished rice. Rice hulls (a and b), husked rice (c and d), bran (e and e'), and polished rice (f and f') were 1 and observed under UV (a, c, e, and f) and bright-field (b, d, e', and f') conditions. Other seeds were sown onto sterilized soil and cultivated for 40 days until the plants reached the heading stage. The sheath joints of first leaves (g and g'), coleoptiles (h and h') and roots (i and i') were observed microscopically under UV (g to i) and bright-field (g' to i') conditions. Scale bars, 500 μm (a and c) and 100 μm (g to i and g' to i').

Because rice seeds are usually stored for about 6 months (from October to March) before sowing in Japan, W5 needed to survive at least 6 months on/in the rice seeds. To evaluate the survivability of W5 on/in the seeds, we spray treated rice flowers with W5-GFP-GEN^r^ and observed the seeds harvested from these flowers. After 6 months of seed storage at 25°C, mycelia showing green fluorescence were recovered from the surface of hulls and hulled grains (endosperm and embryo covered with bran, what is called genmai in Japanese) derived from W5-GFP-GEN^r^-treated flowers on potato sucrose agar containing Geneticin (PSA-GEN; Fig. 3a to e). In contrast, mycelia producing green fluorescence were not observed on the polished rice (the portion that is usually eaten as white rice without bran; hakumai in Japanese) from PSA-GEN-treated seeds (Fig. 3f and f').

Next, we sowed seeds that had been stored for 6 months at 25°C and observed the resulting rice seedlings using a fluorescence microscope. Green fluorescent mycelia were observed on the roots, coleoptiles, and the sheath joints of the leaves (Fig. 3g to i). Collectively, these results indicated that W5-GFP-GEN^r^ can colonize and survive on/in rice seeds for at least 6 months. More importantly, the dynamics of W5 colonization in rice plant tissues resemble those of the bakanae pathogen, F. fujikuroi (21).

### Visualization of the inhibitory effects of W5 against the bakanae pathogen on seedlings generated from seeds soaked in W5 and the pathogen bud-cell suspensions.

To visualize the inhibitory effects of W5 against the bakanae pathogen on rice seedlings, we employed W5-GFP-GEN^r^ and Ff-RFP-HYG^r^ (a restriction enzyme-mediated integration [REMI] transformant of Miyagi 92-10-expressing red fluorescent protein [RFP]) to produce the bakanae pathogen expressing red fluorescent protein (21). We soaked seeds colonized by W5-GFP-GEN^r^ in the bud-cell suspension of Ff-RFP-HYG^r^ under normal pressure, a process that aimed to reproduce how transmission of bakanae pathogen occurs in seed-soaking containers or nursery trays.

**FIG 4:**
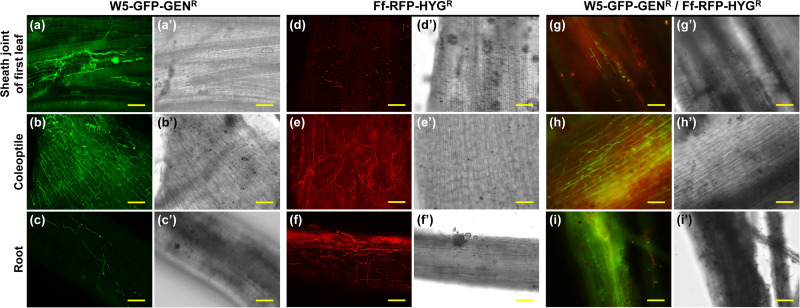
Fluorescence imaging of nonpathogenic Fusarium commune W5-GFP-GEN^r^ and F. fujikuroi Ff-RFP-HYG^r^ on rice (Oryza sativa) seedlings. Twenty-one-day-old plants were generated from seeds that had been vacuum inoculated with bud-cell suspensions of W5-GFP-GEN^r^ (a to c and g to i) or water (d to f), followed by soaking in bud-cell suspensions of Ff-RFP-HYG^r^ (d to i) or water (a to c). Roots, coleoptiles, and the sheath joints of first leaves were observed using a fluorescence microscope. (Panels a' to i' show the bright-field condition.) Scale bars, 100 μm.

When we observed 28-day-old seedlings generated from the seeds treated only with W5-GFP-GEN^r^, we detected W5-GFP-GEN^r^ mycelia at a high frequency (over 50% of all observed plants) on roots, coleoptiles, and the sheath joints of the first and second leaves (Fig. 4a to c). W5-GFP-GEN^r^ was found in fewer than 50% of the plants on the sheath joint of the third leaf and was not found in the fourth or upper leaves (Table 2). The dynamics of W5 on rice seedlings generated from seeds vacuum inoculated with W5 were almost identical to those of W5 on seedlings generated from seeds that developed from flowers spray treated with W5.

**TABLE 2:** Frequency of nonpathogenic Fusarium commune W5-GFP-GEN^r^ and F. fujikuroi Ff-RFP-HYG^r^ infections on rice (Oryza sativa) seedlings grown from seeds dipped into a fungal suspension

Location on plant^*b*^	Level of infection with indicated spray treatment/inoculant (observed fluorescence):			
	W5-GFP-GEN^r^ (water [GFP])	W5-GFP-GEN^r^ (Ff-RFP-HYG^r^ [GFP])	W5-GFP-GEN^r^ (Ff-RFP-HYG^r^ [RFP])	Water (Ff-RFP-HYG^r^ [RFP])
Sheath joint of 4th leaf	−^*a*^	−	−	−
Sheath joint of 3rd leaf	+	+	−	−
Sheath joint of 2nd leaf	++	++	−	+
Sheath joint of 1st leaf	++	++	−	+
Coleoptile	++	++	+	++
Root	++	++	+	++

a −, no fluorescence observed; +, fluorescence was observed in 50% or less of plant tissues; ++, fluorescence was observed in more than 50% of plant tissues.

b Root, coleoptile, and leaves of 8 to 11 plants were observed for each treatment.

On the roots and coleoptiles of 28-day-old seedlings generated from seeds soak inoculated only with Ff-RFP-HYG^r^, the mycelia of Ff-RFP-HYG^r^ were frequently (over 50% of the plants) observed (Fig. 4e and f). We also detected Ff-RFP-HYG^r^ at a lower frequency (less than 50%) on sheath joints of the first and the second leaves (Fig. 4d) but not on the third or upper leaves (Table 2). These dynamics of F. fujikuroi were almost identical to those of W5 mentioned above and to those reported in earlier studies (7, 9).

In contrast, when Ff-RFP-HYG^r^ was inoculated on seeds that were pretreated with W5-GFP-GEN^r^, we observed mycelia of Ff-RFP-HYG^r^ on the roots and coleoptiles in the seedlings at a lower frequency than with a single inoculation of Ff-RFP-HYG^r^ (Fig. 4h and i and Table 2). It is noteworthy that Ff-RFP-HYG^r^ was not detected on the sheath joints of leaves, whereas mycelia of W5-GFP-GEN^r^ were frequently found in the same tissues (Fig. 4g and Table 2).

We also found a lower frequency of Ff-RFP-HYG^r^ on coleoptiles and roots grown from W5-GFP-GEN^r^-treated seeds than those derived from water-treated seeds. These results demonstrated that W5 inhibited the colonization of F. fujikuroi on nursery plants.

### Visualization of the inhibitory effects of W5 against the bakanae pathogen on rice flowers.

As described above, we found that spray treatment of rice flowers with W5 controlled the bakanae pathogen at two steps during the disease cycle, seedling-to-seedling transmission in nursery trays and flower infection with the spores. Thus, we examined the inhibitory effects of W5 against the bakanae pathogen during coinfection of rice flowers by microscopic observation. We sprayed the bud-cell suspensions of W5-GFP-GEN^r^ and Ff-RFP-HYG^r^ sequentially at intervals of 10 min onto the rice flowers and observed the dynamics of W5-GFP-GEN^r^ and Ff-RFP-HYG^r^ with a fluorescence microscope until 21 days after spraying.

**FIG 5:**
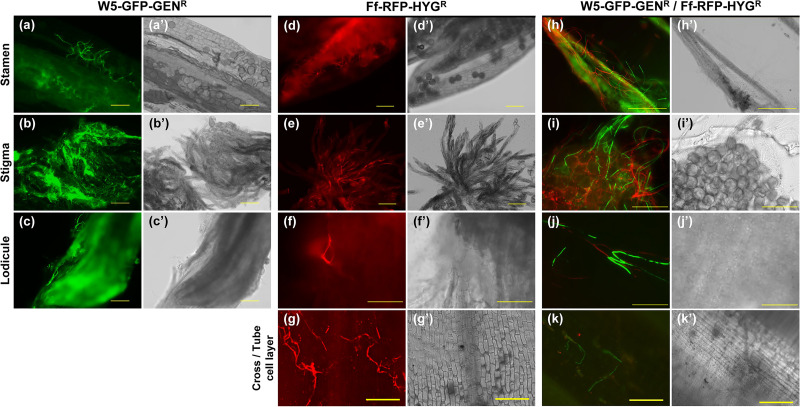
Fluorescence imaging of nonpathogenic Fusarium commune W5-GFP-GEN^r^ and F. fujikuroi Ff-RFP-HYG^r^ on rice (Oryza sativa) flowers. Flowers were sprayed with bud-cell suspensions of W5-GFP-GEN^r^ (a to c and h to k) or water (d to g). After a 10-min interval, flowers were sprayed with bud-cell suspensions of Ff-RFP-HYG^r^ (d to k) or water (a to c). After further incubation for 3 days (a to g) or 5 days (h to k), stamen (a, d, and h), stigmata (b, e, and i), lodicules (c, f, and j), and cross/tube cell layers (g and k) were observed using a fluorescence microscope. (Panels a' to k' show the bright-field condition.) Scale bars, 100 μm.

Red fluorescence was observed in the anthers, stigmata, lodicules, cross cells, and tube cells of Ff-RFP-HYG^r^ single-inoculated flowers (Fig. 5d to g; Table 3; Fig. S8). Similar to our results, Kagiwata (42) reported that F. fujikuroi colonized stigmata and stamens at 48 h postinoculation after dipping flowers in a conidial suspension of F. fujikuroi. Later, mycelia extended to an ovary and proliferated there. Thus, our flower-spraying method is suitable for observing these fungal activities on rice flowers. On flowers spray treated only with W5-GFP-GEN^r^, green fluorescence was frequently observed in the anthers, stigmata, lodicules, pericarp epidermis, and sometimes in the cross/tube cell layers and testae (Fig. 5; Table 3).

**TABLE 3:** Frequency of nonpathogenic *Fusarium commune* W5-GFP-GEN^r^ and F. fujikuroi Ff-RFP-HYG^r^ on rice (Oryza sativa) flowers sprayed with a fungal suspension

Location on plant^*b*^	Level of infection with indicated spray treatment/inoculant at dpi and observed fluorescence^*a*^:																							
	W5-GFP-GEN^r^ (water)						W5-GFP-GEN^r^ (Ff-RFP-HYG^r^)												Water (Ff-RFP-HYG^r^)					
	1	3	5	7	14	21	1		3		5		7		14		21		1	3	5	7	14	21
	G	G	G	G	G	G	G	R	G	R	G	R	G	R	G	R	G	R	R	R	R	R	R	R
Anther	+	++	++	++	++	++	+	+	++	+	++	++	++	++	++	NT	NT	NT	+	++	++	++	++	++
Stigma	+	++	++	++	++	++	+	+	++	++	++	++	++	++	++	++	++	+	+	++	++	++	++	++
Lodicule	+	++	++	++	++	++	+	+	+	+	++	+	+	+	++	+	++	+	+	++	++	++	++	++
Epidermis of ovary	+	++	++	++	++	++	+	+	−	−	+	−	+	−	+	+	++	+	+	++	++	++	++	++
Cross/tube cell layer	NT	−	+	+	−	−	NT	NT	−	−	+	−	+	−	−	−	−	−	NT	−	+	+	−	−
Testa and perisperm	NT	NT	+	−	−	−	NT	NT	NT	NT	−	−	−	−	−	−	−	−	NT	NT	−	−	−	−
Endosperm	NT	NT	−	−	−	−	NT	NT	NT	NT	−	−	−	−	−	−	−	−	NT	NT	−	−	−	−
Embryo	NT	NT	NT	−	−	−	NT	NT	NT	NT	NT	NT	−	−	−	−	−	−	NT	NT	NT	−	−	−

a G, green fluorescence of GFP; R, red fluorescence of RFP; +, fluorescence was observed in 50% or less of plant tissues; ++, fluorescence was observed in more than 50% of plant tissues; −, no fluorescence observed; NT, not tested.

b Root, coleoptile, and leaves of 8 to 11 plants were observed for each treatment.

Remarkably, by observing flowers spray treated with W5-GFP-GEN^r^ and inoculated with Ff-RFP-HYG^r^, the red fluorescence indicative of Ff-RFP-HYG^r^ infection was not observed within the cross and tube cell layers (Fig. 5k), and the frequency of red fluorescence was reduced in lodicules and the ovary epidermis (Fig. 5j; Table 3). These results suggested that W5 protected the epidermis from entry of the bakanae pathogen. Although Ff-RFP-HYG^r^ was strongly affected by the presence of W5-GFP-GEN^r^ in terms of its growth within rice flowers at least, no penetration or degradation of Ff-RFP-HYG^r^ by W5-GFP-GEN^r^ was observed. These observations indicated that W5 has biocontrol activity of bakanae disease that is different from that of Trichoderma asperellum SKT-1 or Talaromyces flavus SAY-Y-94-01, both of which are commercially used biocontrol agents and are reported to be mycoparasites of F. fujikuroi (19, 43).

Populations of W5-GFP-GEN^r^ on lodicules, ovary epidermis, and cross/tube cell layers were reduced by Ff-RFP-HYG^r^ at an earlier stage (Table 3); however, the reduction was much smaller than that of Ff-RFP-HYG^r^ by W5-GFP-GEN^r^ (Table 3), and Ff-RFP-HYG^r^ disappeared at 21 days postinoculation (dpi) (Table 3). This finding indicated that W5 competed with F. fujikuroi on rice flowers and inhibited F. fujikuroi’s colonization; however, further investigation is needed to determine how this inhibition is accomplished. There are several reports that nonpathogenic fusaria induce disease resistance in plants (44). As presented in Fig. 1, seed treatment with W5 sometimes reduced the growth of rice plants. Growth reduction is often observed when disease resistance is induced in plants by biocontrol agents (45) or by plant activators (46), suggesting that W5 possibly induced resistance in rice. Further investigation is necessary to explain in the detail the mode of action of W5.

W5 proved to colonize rice flowers, colonize seeds, and elongate mycelia on seedlings. W5 also can reduce the population of F. fujikuroi in/on rice plants, probably by competition. These observations likely reflect analogous colonization/infection processes between the nonpathogenic W5 and pathogenic F. fujikuroi.

W5 reached the bran of grains when flowers were sprayed with bud-cell suspensions but did not reach the endosperm or embryo (Table 3), suggesting that if W5 was sprayed on rice flowers for food production, contamination of W5 in the edible portions of rice grains is of no concern.

## DISCUSSION

Rice bakanae disease caused by F. fujikuroi is one of the most destructive diseases for rice cultivation. Seed disinfection by chemicals, biofungicides, or hot water is currently the only effective methods to control this disease; however, these methods have limited efficacy when a seed stock is deeply contaminated or latently infested with F. fujikuroi (10, 30). Therefore, a new method that can reduce the number of infested seeds, namely, a means of suppressing flower infection by F. fujikuroi, is required for stable control of bakanae disease.

In this study, we demonstrated that treating rice flowers with biocontrol agents is able to control bakanae disease in plants of the next generation. We isolated 106 fusaria from Vietnamese and Japanese rice plants and selected four nonpathogenic fusaria isolates that possessed biocontrol activity against bakanae disease. Among the four isolates, two were identified as F. commune, and the others were F. proliferatum. Spray treatment of flowers with the nonpathogenic F. commune isolates W3 and W5, thereby mimicking the disease cycle of F. fujikuroi, presented excellent activity in reducing the number of bakanae-infested seeds in a field experiment. We propose that this may be a novel treatment method for introducing biocontrol agents capable of suppressing bakanae disease. We visualized W5-bakanae pathogen interaction on/in rice plants using transformants of W5 and F. fujikuroi expressing GFP and RFP, respectively, and showed that W5 inhibited hyphal extension of F. fujikuroi on/in rice flowers and seedlings possibly by competition. Our study also demonstrated that W5 sprayed on flowers survived on/in rice seeds for at least 6 months.

*Fusarium* species are known to produce many kinds of secondary metabolites, including mycotoxins, for example, fumonisins, trichothecenes, and zearalenone (41, 47). F. proliferatum, the species identity of two (a25 and a29) of the four isolates we selected, is known to be a producer of fumonisin. Due to the possibility that a25 and a29 might be able to produce mycotoxins, we did not proceed with field trials for these two isolates. F. commune was newly identified and found to be a separate species from F. oxysporum in 2003 (48). F. commune is known to be one of the predominant *Fusarium* species in rice grains (49). The ability of F. commune to produce mycotoxins is still unclear, but this species seems closely related to F. oxysporum. F. oxysporum is known to produce some secondary metabolites such as beauvericin and fusaric acid; however, most isolates of the F. oxysporum species complex are considered to be nontoxigenic and nonproducers of trichothecenes, fumonisins, and zearalenone (41, 47). We have also obtained chromosome-scale (meansing almost telomere-to-telomere quality) whole-genome sequence data of W3 and W5 (data not shown), which indicated that both W3 and W5 have no gene or no gene cluster associated with the production of harmful mycotoxins, such as fumonisins, zearalenone, or deoxynivalenol. These data will be shown in another report. According to these findings, we surmise that F. commune W3 and W5 do not produce harmful mycotoxins. Furthermore, our biocontrol strategy, spraying nonpathogenic fusaria onto flowers, should be used only for seed production and not for food production. Although our proposed method for treating biocontrol agents is considered to have a shallow risk to food, applying any biocontrol product based on these strains should be realized only after thorough risk analysis regarding the application area’s surroundings.

There are many reports of using seed treatments with biocontrol agents against seed-borne diseases (50); however, there have been no studies that report application of biocontrol agents to plant flowers to control seed-borne diseases. Furthermore, heavily infested seeds are known to be difficult to disinfect with conventional chemical or hot water seed treatments (30); however, our findings suggested that treatment of flowers with nonpathogenic fusaria reduced the number of heavily infested seeds. Therefore, spraying flowers with microbial agents can stably control seed-borne diseases and can be an alternative to seed disinfection.

Rice ovaries remain and cover the seed as testae until germination (51). Therefore, biocontrol agents adhering to the ovary of flowers can remain on the seed testa and be transmitted to the next plant generation. The seeds of other *Poaceae* crops and vegetables, such as spinach, lettuce, and carrot, are also covered with testa originated from ovaries, and our method can be applied for those plants.

## MATERIALS AND METHODS

### Plant materials and bakanae pathogen.

Rice (Oryza sativa subsp. *japonica*) cv. Tanginbouzu, which has a mutation in the kaurene oxidase gene causing reduced gibberellin biosynthesis (52) and a semidwarf phenotype, was used in all experiments. Rice seeds (cv. Koshihikari) naturally infested with F. fujikuroi were used as inocula in paddy field trials. F. fujikuroi, the bakanae pathogen (isolate Miyagi 92-10), was obtained from the Kureha Corporation, Research Center, Iwaki, Fukushima, Japan. Ff-RFP-HYG^r^, a restriction enzyme-mediated integration (REMI) transformant of Miyagi 92-10-expressing red fluorescent protein (RFP) (21), was used to analyze the dynamics of the bakanae pathogen in rice seeds and plant tissues. Ff-RFP-HYG^r^ showed bakanae symptoms indistinguishable from those caused by the progenitor strain F. fujikuroi Miyagi 92-10 (21).

### Plant and fungal growth conditions.

For every plant assay, rice seeds were soaked in water, solvent, or fungal suspension at 15°C for 4 days and then incubated at 30°C for 24 h in the dark to hasten germination. Germinated seeds were sown in sterilized soil (Kumiai Nippi Engei soil no. 1; Nihon Hiryo, Tokyo, Japan), and the seedlings were maintained in a growth chamber (28°C:25°C, 12 h:12 h, light:dark) or a glasshouse (ca. 28°C, natural light conditions). Every fungal isolate was maintained and incubated at 28°C on PSA (0.5% [wt/vol] sucrose and 1.5% [wt/vol] agar in potato extract) medium.

### Isolation of nonpathogenic fusaria from rice tissues.

All Vietnamese fusaria used in this study were isolated from bakanae-diseased rice plants growing in An Giang, Hau Giang, Soc Trang, Tien Giang, and Vinh Long provinces and Can Tho city in 2008. Japanese fusaria used in this study were isolated from healthy or bakanae-diseased rice plants sampled from the bakanae-outbreaking paddy fields in Osato and Toyoma, Miyagi Prefecture, and Inakadate and Kuroishi, Aomori Prefecture, in 2011. Rice leaf blades were cut into ca. 2-cm-long pieces and surface sterilized with 70% (vol/vol) ethanol and 1% (vol/vol) sodium hypochlorite. The sterilized leaf segments were placed on 1% (wt/vol) water agar plates and incubated at 28°C under dark conditions for 1 week. Mycelia grown from the segments were transferred to fresh PSA plates and maintained as described above.

### Preparation of bud-cell suspensions of the pathogen and isolated fusaria.

Fusaria are known to produce a large number of yeastlike spores called bud cells in shaking liquid culture that can be easily suspended in water (53). To prepare a bud-cell suspension of F. fujikuroi or *Fusarium* isolates, fungi were cultured in potato sucrose broth (PSB; 0.5% [wt/vol] sucrose in potato extract) for 5 days by reciprocal shaking at 120 strokes/min. The bud cells were filtered through a double layer of gauze to remove mycelia. The filtrates were centrifuged at 1,600 × *g* for 10 min, and the precipitated bud cells were suspended in sterilized water.

### Selection of biocontrol agents.

Rice seeds infested with bakanae pathogen were prepared as described in Kato et al. (21) and used for biocontrol activity assays.

Biocontrol agents were selected in two steps. In the primary selection, all 106 isolates were tested on a small scale using plastic ice cube trays as nursery pots. Before sowing, the infested seeds were soaked in a 5-fold diluted culture filtrate of each isolate. For the controls, infested seeds were soaked in a 200-fold diluted solution of Eco-hope (Kumiai Chemical Industry, Tokyo, Japan; containing 1 × 10^8^ CFU/ml Trichoderma asperellum [registered as Trichoderma atroviride under Agricultural Chemicals Regulation Act in Japan] SKT-1), a 200-fold diluted solution of Techlead-C Flowable (Kumiai Chemical Industry; containing 5% ipconazole [Kureha] and 3% copper), or sterilized water (control). Seeds were sown onto the sterilized soil in each cell of the plastic ice cube tray (20 seeds per cell; three cells per isolate) and grown in an incubator for 14 days. After incubation, the disease incidence rate was calculated.

The secondary selection was performed at a larger scale using plastic pots (200 ml). Before sowing, the infested seeds were soaked in a bud-cell suspension (1.0 × 10^6^ spores/ml) of each selected isolate. All seeds were then sown into sterilized soil in plastic pots (ca. 70 seeds per pot) and maintained in a glasshouse for 14 days. After incubation, the disease incidence rate was calculated.

### DNA extraction, amplification, and sequencing.

Genomic DNA of four selected isolates (a25, a29, W3, or W5) was extracted from mycelia as described in Saitoh et al. (54) and used for PCR amplification of the rDNA internal transcribed spacer region (ITS) with primers ITS1 and ITS4 (55) and the intergenic spacer region (IGS) with primers FIGS11 and FIGS12 (56). Each amplicon was purified with ExoSAP-IT (Thermo Fisher Scientific, Waltham, MA, USA) and used for sequencing reactions as described in Platt et al. (57). Capillary sequencing was carried out using an Applied Biosystems 3130xl DNA analyzer (Thermo Fisher Scientific). The generated sequence data were trimmed using GENETYX-MAC version 11.2.1 (Genetyx Corporation, Tokyo, Japan) and used to identify each isolate with BLASTN searches.

### Spray treatments of biocontrol agents onto flowers of growth chamber-grown plants.

Healthy rice seeds were sown in sterilized soil in plastic pots (three seeds per pot), and plants were grown in the growth chamber until harvest. At anthesis, which lasts for about 10 days, rice flowers were sprayed four times (1st, 3rd, 5th, and 7th days after first flowering) with a bud-cell suspension (1.0 × 10^5^ spores/ml; 5 ml for each panicle of rice) of each of the selected *Fusarium* isolates (a25, a29, W3, or W5). The seeds were harvested from the sprayed flowers ca. 50 days after spraying and were inoculated by soaking in a bud-cell suspension (1.0 × 10^3^ spores/ml) of F. fujikuroi until the seeds were sown. Seed sowing, incubation of plants, and evaluation of disease incidence were performed in the same way as described for the secondary selection of the isolates.

### Field trials of spray treatments with biocontrol agents onto flowers.

Field trials were performed in paddy fields at the Honmachi Field Science Center of Tokyo University of Agriculture and Technology (TUAT), Fuchu, Tokyo, Japan. Four experimental plots, each with 144 plants in 2012 or 100 plants from 2013 to 2015, were prepared for each treatment. Healthy 30-day-old rice seedlings (cv. Tanginbouzu) were transplanted on 28 May 2012, 30 May 2013, 27 May 2014, or 25 May 2015. Plots transplanted with bakanae-infected seedlings (cv. Koshihikari) were distributed among the experimental plots to supply inocula (Fig. S4 in the supplemental material). At anthesis, rice flowers were sprayed with a bud-cell suspension (1.0 × 10^5^ spores/ml; 5 ml for each panicle of rice) of selected *Fusarium* isolates (W3 or W5). Flower treatments were carried out twice in each year on the following dates: 24 and 27 August 2012, 26 and 27 August 2013, 25 and 29 August 2014, and 27 and 31 August 2015. The seeds were harvested in October and used after being dried and threshed.

### Evaluation of seed quality and assessment of bakanae control in field trials.

The quality of seeds obtained from sprayed flowers produced in the paddy field was evaluated according to conventional procedures (58) with modification. To evaluate germination vigor and percentage, seeds were placed on sterilized filter paper in glass petri dishes, incubated at 25°C under dark conditions, and irrigated with sterilized water appropriately. The disease incidence rate was evaluated as described for the growth chamber trial methods.

### Plasmid construction.

Vector pMD-GEN was constructed by inserting an EcoRI-digested 1.7-kb fragment of pMK412 (19), containing the *egfp* cassette driven by the *TEF* promoter and terminated by the *gla* terminator, into pII99 (59) digested with the same restriction enzyme (Fig. S6A).

### Production of a GFP-expressing transformant of W5 using the REMI method.

In this study, REMI was used to produce W5 transformants following the procedures described in Watanabe et al. (19). Protoplasts of W5 (ca. 1.0 × 10^7^ cells) were mixed with a plasmid mixture containing 35 μg of pMD-GEN digested by 100 U BglII (New England Biolabs, Ipswich, MA, USA) without inactivating the restriction enzyme. Transformed protoplasts were regenerated on the regeneration medium (RM; 1 M sucrose, 0.1% [wt/vol] yeast extract, and 1% [wt/vol] agar in distilled water) containing 100 μg/ml of Geneticin (G 418 disulfate salt; Merck, Darmstadt, Germany). A colony whose mycelia presented the strongest and most stable green fluorescence under 460 to 495 nm blue light was selected and maintained on PSA containing 100 μg/ml of Geneticin (PSA-GEN).

### Reisolation and observation of W5-GFP-GEN^r^ from rice seeds and plants generated from sprayed flowers.

Flowers of healthy rice plants were sprayed with a bud-cell suspension of W5-GFP-GEN^r^ (1.0 × 10^6^ spores/ml; 3.0 ml for each panicle of rice) or sterilized water (control; 3.0 ml for each panicle) in a cabinet, and the plants were maintained in a growth chamber. Rice seeds obtained from those flowers were stored at 25°C for 6 months, after which time seeds were divided into rice hulls and hulled grains using a TR-250 automatic rice husker (Kett Electric Laboratory, Tokyo, Japan). The hulls and hulled grains were separately placed on PSA-GEN. In addition, some of the hulled grains were further polished using a Pearlest grain polisher (Kett Electric Laboratory) and separated into bran and milled rice grain. The bran and milled rice grain were separately distributed on PSA-GEN, incubated for 5 days at 28°C, and observed macroscopically under a UV lamp. Some additional seeds were sown in sterilized soil and maintained in the growth chamber for 40 days. After cultivation, plants were separated into roots, coleoptiles, and the sheath joint of the leaves. Subsequently, each part of the plant was observed using a fluorescence microscope (BX53; Olympus, Tokyo, Japan). Tissues from 10 plants were used for the observations. Excitation of the fluorescent proteins was observed with a mirror unit U-FBNA (470 to 495 nm; Olympus) for GFP or U-FGW (530 to 550 nm; Olympus) for RFP using a mercury arc lamp. Emission was captured with a barrier filter, BA510-550 (under 470 nm and over 550 nm) or BA575IF (under 530 nm), which was included in a mirror unit, respectively.

### Fluorescence imaging of fungal interactions in rice nursery plants.

To prepare the W5-GFP-GEN^r^-treated rice seeds, healthy seeds were soaked in a bud-cell suspension of W5-GFP-GEN^r^ under vacuum conditions as described in Kato et al. (21). The seeds (6 g) were soaked in 12 ml of a bud-cell suspension (1.0 × 10^3^ spores/ml) of Ff-RFP-HYG^r^ or sterilized water (control) until sowing. After sowing, the seedlings were maintained in a growth chamber for 28 days. After careful harvesting, each seedling was separated into roots, coleoptiles, and first, second, third, and fourth leaves (sheath and blade) and observed using a fluorescence microscope.

Flowers of rice plants grown from the healthy seeds were sprayed with fungal bud-cell suspension in the following three treatments: W5-GFP-GEN^r^ (1.0 × 10^6^ spores/ml, 3.0 ml for each panicle) and, 10 min later, water (3.0 ml); with water (3.0 ml) and, 10 min later, Ff-RFP-HYG^r^ (1.0 × 10^6^ spores/ml); or with W5-GFP-GEN^r^ (1.0 × 10^5^ spores/ml) and, 10 min later, Ff-RFP-HYG^r^ (1.0 × 10^5^ spores/ml). The interior and exterior tissues of sprayed flowers (Fig. S7) were observed with a fluorescence microscope chronologically at 1, 3, 5, 7, 14, and 21 days after spraying.

### Statistical analysis.

The Tukey’s test was conducted using the R-package multcomp (60), and a *P* value lower than 0.05 was considered a significant difference. Different letters indicate significant differences between the treatments in Table 1, Fig. 1, and Fig. 2.

### Data availability.

The sequences obtained in this study are available with accession numbers LC516582 to LC516590 at the DDBJ/EMBL/GenBank database.

## Supplementary Material

Supplemental file 1
