# Supplementary material for: Spray Application of Nonpathogenic Fusaria onto Rice Flowers Controls Bakanae Disease (Caused by Fusarium fujikuroi) in the Next Plant Generation
Source: Appl Environ Microbiol. 2021 Jan 4;87(2):e01959-20. doi: 10.1128/AEM.01959-20 (PMC7783350; doi:10.1128/AEM.01959-20)
Supplement: Supplemental file 1 [file AEM.01959-20-s0001.pdf]

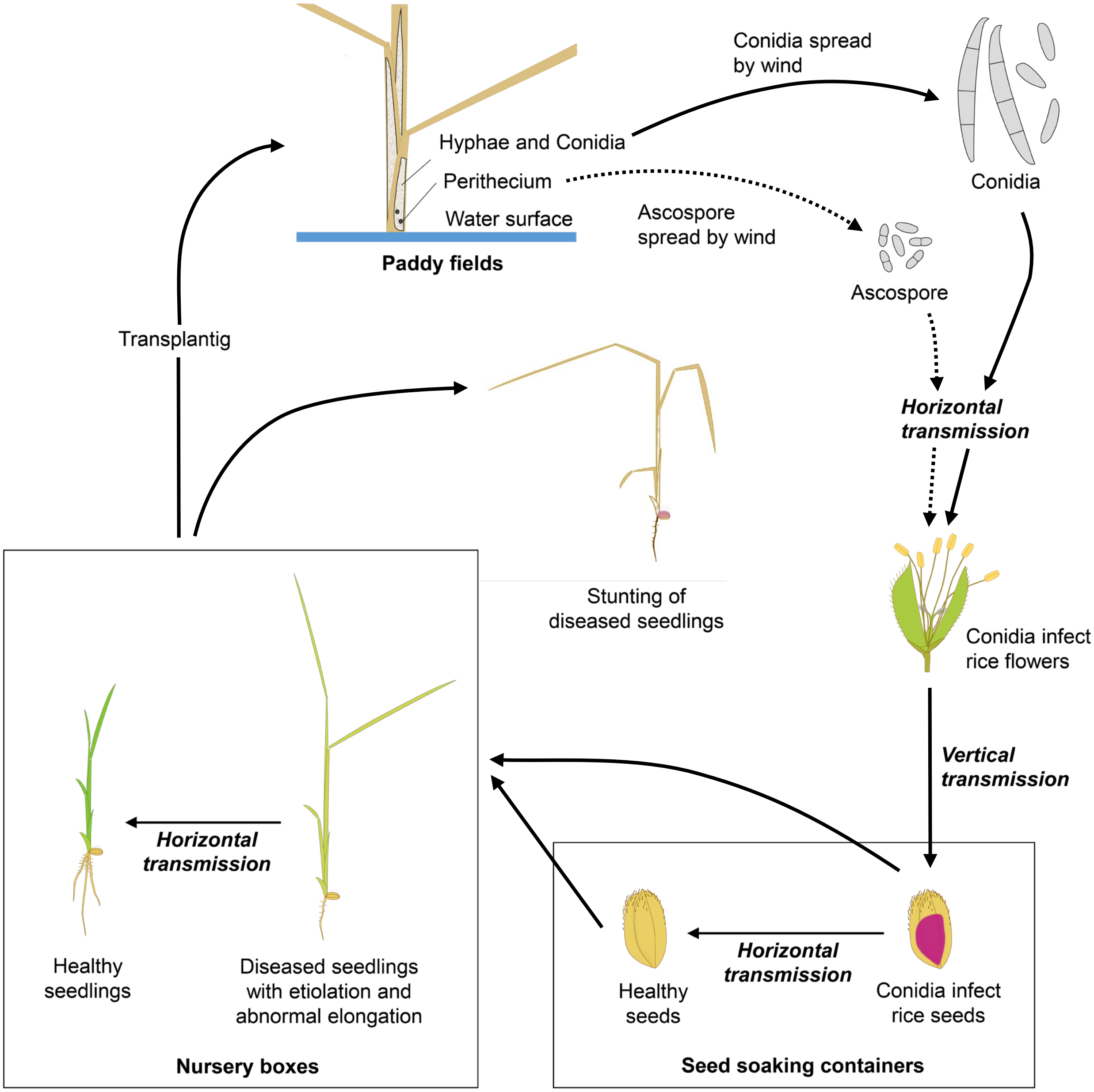

FIG S1 Disease cycle of bakanae of rice (*Oryza sativa*) caused by *Fusarium fujikuroi*.

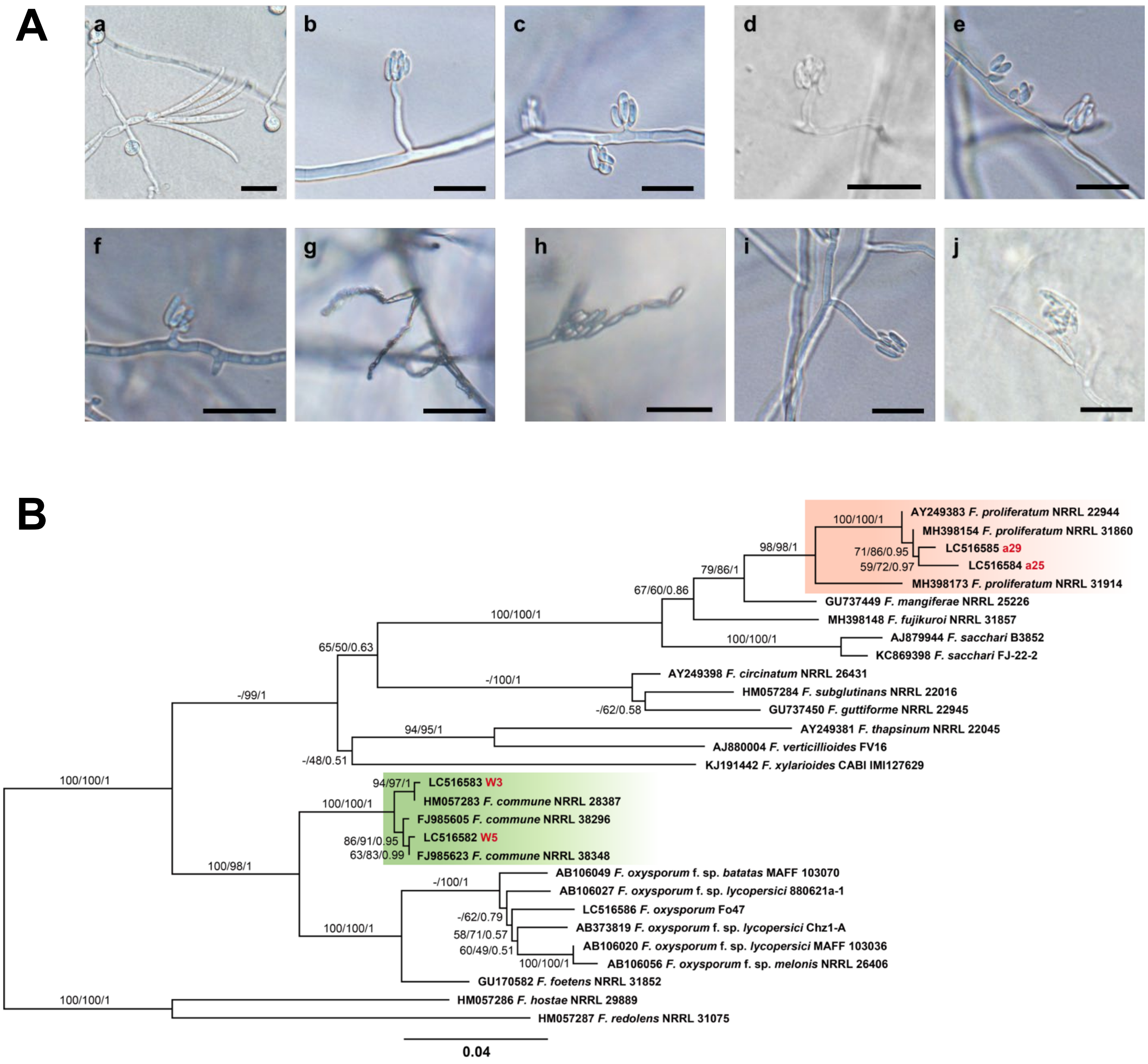

FIG S2 Morphological and phylogenetic characteristics of selected biocontrol candidates. **A)** *Fusarium commune* W5 (a–c), W3 (d,e), *F. proliferatum* a25 (f,g) and a29 (h–j) after two-weeks incubation on SNA (Spezieller nährstoffarmer agar) (1) at 28°C. (a) Macroconidia and chlamydospores; (b–f,i) microconidia in false heads; (g,h) chains of microconidia; (j) macroconidia and microconidia. Scale bars, 20 µm. **B)** Maximum likelihood (ML) tree based on the sequences of the intergenic spacer (IGS) region of *Fusarium* species. The sequences of *Fusarium redolens* NRRL 31075 and *F. hostae* NRRL 29889 were used as outgroups. Node supports given for maximum-parsimony bootstrap values, maximum-likelihood bootstrap values, and Bayesian posterior probabilities are shown on each branch. Detailed methods are described in supporting information Materials and Methods.

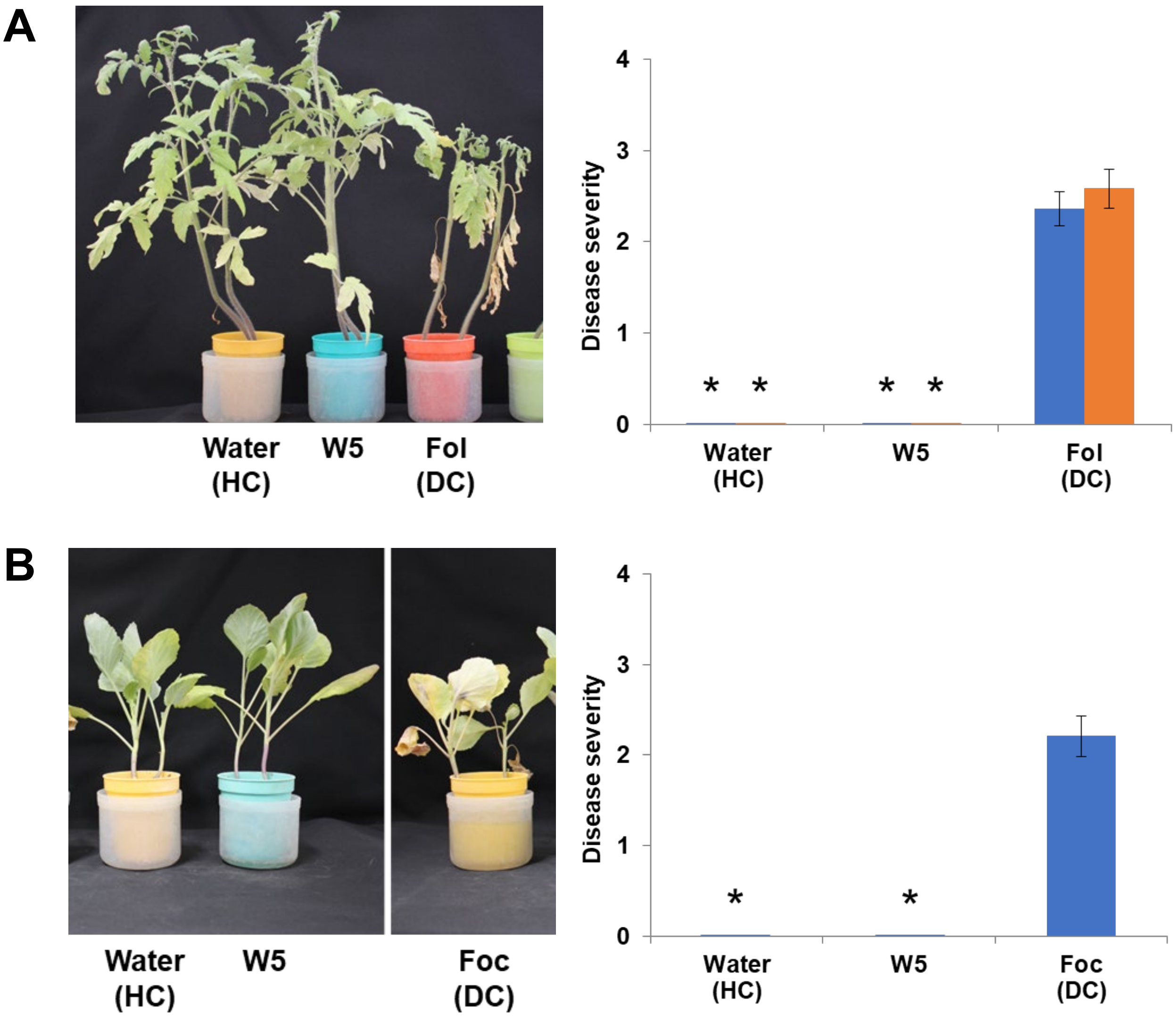

FIG S3 Pathogenicity test of a biocontrol agent *Fusarium commune* W5. Asterisks in bar graphs indicate significant differences of disease severity between water/W5-inoculated and pathogen-inoculated (DC) plants by T-test (P<0.05). **A)** Three-week-old tomato plants (cv. Momotaro; susceptible to *F. oxysporum* f. sp. *lycopersici* (Fol) race 2; Takii & Co., Kyoto, Japan) were inoculated with bud-cell suspensions (1.0×10^7 bud-cells/ml, 1 ml for each plant) of W5 or Fol isolate 880621a-1 (race 2) (DC), or sterile distilled water (HC) by drenching. Four weeks later, the external (blue) or internal (orange) symptoms of each plant were evaluated as described in Inami et al (2). **B)** Three-week-old cabbage plants (cv. Shikidori; susceptible to *F. oxysporum* f. sp. *conglutinans* (Foc); Takii & Co.) were inoculated with bud-cell suspensions (1.0×10^6 bud-cells/ml, 1 ml for each plant) of W5 or Foc isolate Cong:1-1 (DC), or sterile distilled water (HC) by drenching. Two weeks later, the external symptoms of each plant were evaluated as described in Kashiwa et al (3).

A

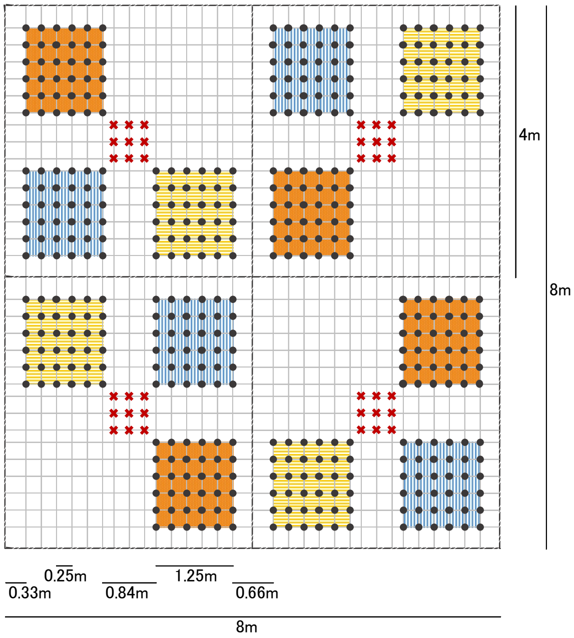

B

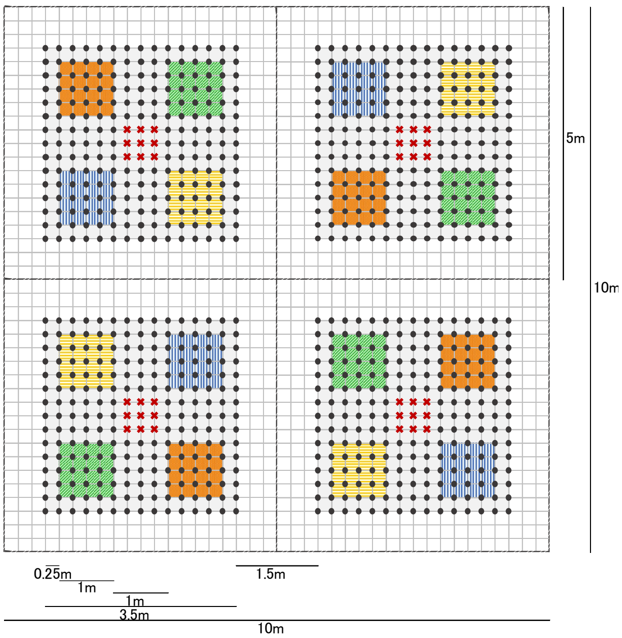

FIG S4 Schematic explanation of test plots in field trial in Honmachi Field Science Center of TUAT in 2012–2015. Each black dot indicates a healthy rice plant (cv. Tanginbouzu) and red x represents a naturally bakanae-infected rice plant (cv. Koshihikari). The area painted in orange, yellow lateral stripes, green diagonal stripes or blue vertical stripes indicate plants which flowers were sprayed with bud-cell suspensions of *Fusarium commune* W5 or W3, spore suspension of Trichoderma, or water, respectively. **A)** in 2012. **B)** in 2013-2015.

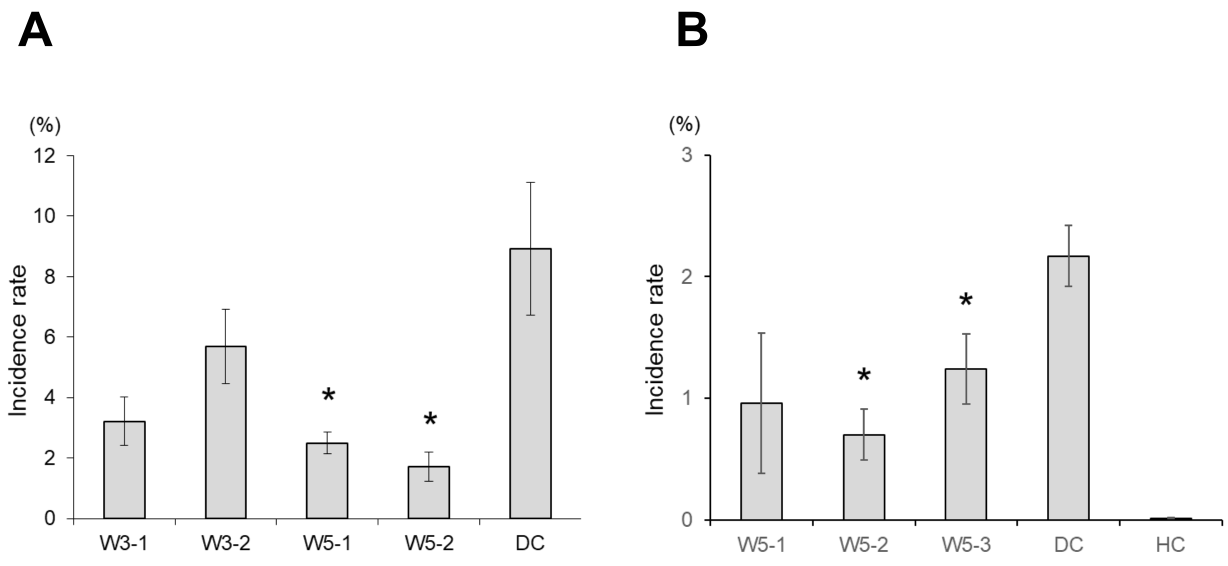

FIG S5 Biocontrol efficacy of spraying flowers with selected non-pathogenic *Fusarium commune* W3 or W5 on bakanae disease in some rice (*Oryza sativa*) cultivars. The incidence rate was calculated as a percentage of the diseased plant number per total plant number for each treatment at 14 days after sowing seeds, which generated from seeds that developed from spray-treated flowers. Asterisks above some bars indicate significant differences of the incidence rate between W3/W5-treated and DC plants by T-test (P<0.05). **A)** Field trial in bakanae-outbreaking paddy fields at Miyagi Furukawa Agricultural Experiment Station using rice plants cv. Hitomebore. At anthesis, flowers were splayed once (W3-1, W5-1) or twice (W3-2, W5-2) with bud-cell suspensions (1.0 × 10^5 bud-cells/ml, 5 ml for each panicle of rice) of W3 or W5. As a diseased control (DC), some flowers were splayed once with sterile distilled water. **B)** Field trial at Aomori Prefectural Industrial Technology Research Center using rice plants cv. Masshigura. At anthesis, flowers were splayed once (W5-1), twice (W5-2) or three times (W5-3) with bud-cell suspensions (1.0 × 10^5 bud-cells/ml, 5 ml for each panicle of rice) of W5. Flowers excluding healthy control (HC) also splayed once with a bud-cell suspension (1.0 × 10^5 bud-cells/ml, 5 ml for each panicle of rice) of a bakanae pathogen.

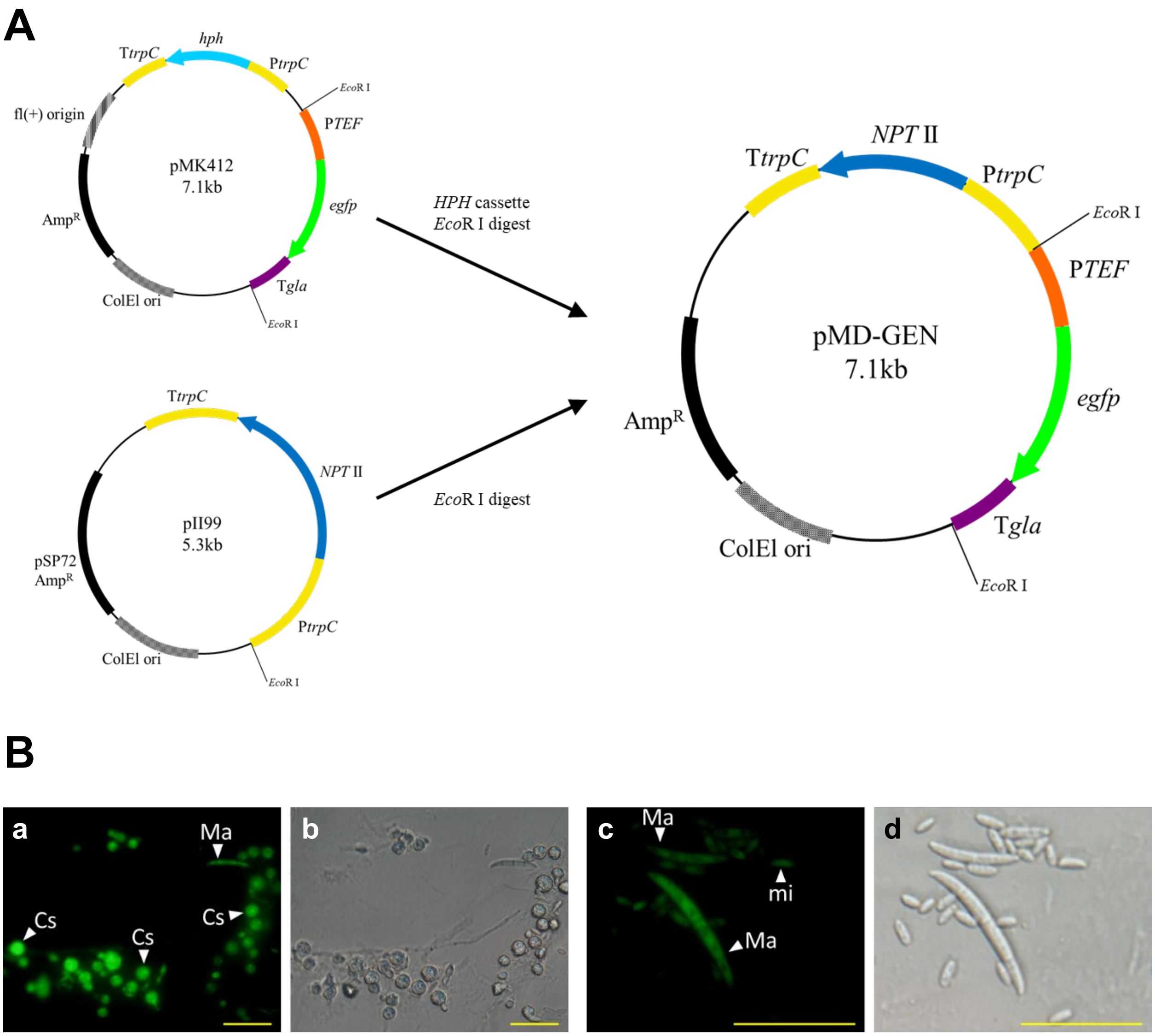

FIG S6 Production of a GFP-expressing transformant W5-GFP-GEN^{R} derived from *Fusarium commune* W5. **A)** Construction of a vector pMD-GEN. Vector pMD-GEN was constructed by inserting an *Eco*R I-digested 1.7-kb fragment of pMK412 (4), containing the *egfp* cassette driven by the *TEF* promoter and terminated by the *gla* terminator, into pII99 (5) digested with the same restriction enzyme. **B)** Green fluorescence of chlamydospores, macroconidia, microconidia of non-pathogenic *F. commune* W5-GFP-GEN^{R} after one-month incubation on PDA at 28°C. Fungal spores were observed microscopically under 460–495 nm UV (a,c) and bright-field (b,d) conditions. Cs, chlamydospores; Ma, macroconidia; mi, microconidia. Scale bars, 20 μm.

A

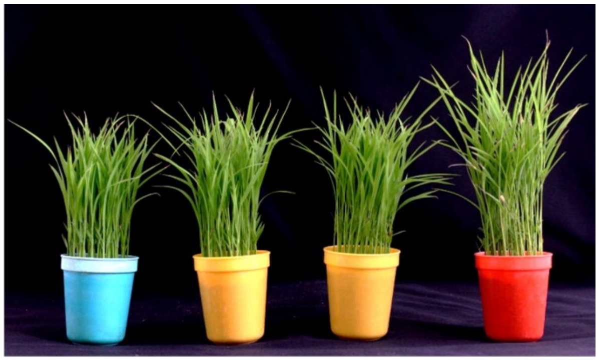

Vacuum infiltration	Water	W5-WT	W5-GFP-GEN^R	Water
Seed soaking	Water (HC)	Ff	Ff	Ff (DC)
Incidence rate (%)	0.0 ^{a}	4.1 ^{a}	3.6 ^{a}	27.3 ^{b}
Prevent value	-	85.1	90.0	0

B

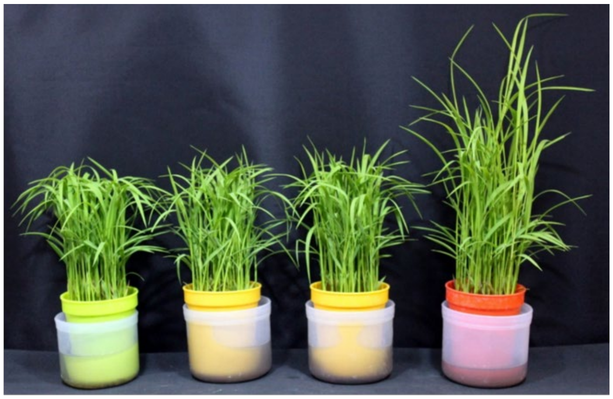

Flower spraying	Water	W5-WT	W5-GFP-GEN^R	Water
Seed soaking	Water (HC)	Ff	Ff	Ff (DC)
Incidence rate (%)	0.0 ^{a}	1.5 ^{a}	3.9 ^{a}	31.0 ^{b}
Prevent value	-	95.1	87.4	0

FIG S7 Biocontrol efficacy of seed or flower treatment with W5-GFP-GEN^R, green-fluorescent transformant of *Fusarium commune* W5, on bakanae disease in rice (*Oryza sativa*) cv. Tanginbouzu. **A)** Healthy seeds were treated with bud-cell suspensions of W5 (W5-WT), W5-GFP-GEN^R or water (HC or DC) by vacuum, then soaked in bud-cell suspensions of bakanae pathogen *F. fujikuroi* Miyagi 92-10 (Ff), or water (HC). 14-day-old plants generated from those seeds were used for evaluation. **B)** Flowers of healthy plants were sprayed with bud-cell suspensions of W5 (W5-WT), W5-GFP-GEN^R or water (HC, DC). Harvested seeds are soaked in bud-cell suspensions of bakanae pathogen (Ff) or water as control (HC). The incidence rate was calculated as a percentage of the diseased plant number per total plant number for each treatment at 14 days after sowing. The preventive value was calculated with the following formula: (Preventive value) = {(Incidence rate of DC) – (Incidence rate for each treatment)} × 100

/ (Incidence rate of DC). In the “Incidence rate (%)” line, different letters indicate significant differences between the treatments by Tukey’s test (P < 0.05).

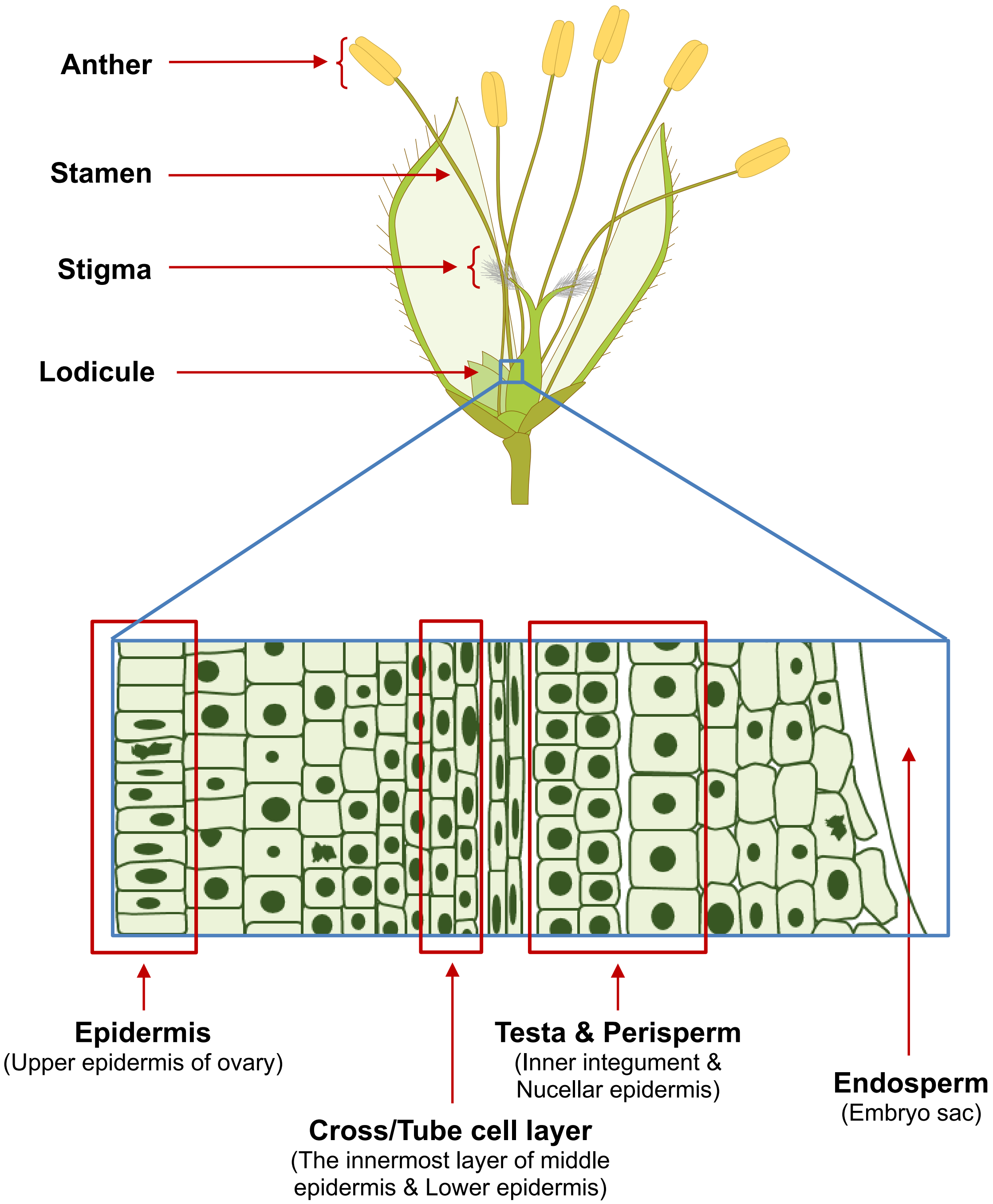

FIG S8 Tissues of rice (*Oryza sativa*) flower. This figure was created with reference to the original drawing by the late Dr. Hoshikawa (6).

TABLE S1 Biocontrol efficacy of seed treatment with 106 fusaria isolates on bakanae disease in rice on a small scale using ice cube trays. “Diseased” in “Condition of source plant” column indicates that the isolate was obtained from a plant tissue with bakanae symptoms, and “Healthy” means the isolate from a symptomless plant tissue. Rice seeds infested with bakanae pathogen were used for biocontrol assays. Three plots for each isolate were used for evaluation and two independent trials were performed. “++” in “Biocontrol efficacy” column indicates that disease incidence rate was lower than 33.4% in both of two trials, “+” indicates that incidence rate was lower than 33.4% in either of trials, and “-” means incidence rate was 33.4% or higher in both trials. In this trial, control plants presented 100.0% disease incidence rate.

Isolate ID	Condition of source plant	Collection place	Biocontrol efficacy
a2	Diseased	An Giang, Vietnam	-
a4	Diseased	An Giang, Vietnam	-
a5	Diseased	An Giang, Vietnam	-
a6	Diseased	An Giang, Vietnam	-
a10	Diseased	Can Tho City, Vietnam	-
a13	Diseased	Can Tho City, Vietnam	-
a16	Diseased	Hau Giang, Vietnam	-
a19	Diseased	Hau Giang, Vietnam	-
a24	Diseased	Tien Giang, Vietnam	-
a25	Diseased	Tien Giang, Vietnam	++
a27	Diseased	Tien Giang, Vietnam	-
a28	Diseased	Tien Giang, Vietnam	-
a29	Diseased	Tien Giang, Vietnam	++
a30	Diseased	Tien Giang, Vietnam	-
a32	Diseased	Vinh Long, Vietnam	-
a33	Diseased	Vinh Long, Vietnam	-
b1	Diseased	Can Tho City, Vietnam	-
b2	Diseased	Can Tho City, Vietnam	-
b3	Diseased	Can Tho City, Vietnam	-
b6	Diseased	Can Tho City, Vietnam	-
b8	Diseased	Can Tho City, Vietnam	-
b9	Diseased	Can Tho City, Vietnam	+
b11	Diseased	Can Tho City, Vietnam	-
b13	Diseased	Can Tho City, Vietnam	-
b14	Diseased	Can Tho City, Vietnam	-
b15	Diseased	Can Tho City, Vietnam	++
b16	Diseased	Can Tho City, Vietnam	-
b19	Diseased	Tien Giang, Vietnam	-
b21	Diseased	Tien Giang, Vietnam	-
b24	Diseased	An Giang, Vietnam	-
b26	Diseased	An Giang, Vietnam	-
b30	Diseased	Hau Giang, Vietnam	-
b36	Diseased	Hau Giang, Vietnam	-
b40	Diseased	Soc Trang, Vietnam	-
b41	Diseased	Soc Trang, Vietnam	-
b43	Diseased	Vinh Long, Vietnam	-

(TABLE S1 cont.)

Isolate ID	Condition of source plant	Collection place	Biocontrol efficacy
B1	Healthy	Osato, Miyagi, Japan	-
B2	Healthy	Osato, Miyagi, Japan	-
O1	Healthy	Osato, Miyagi, Japan	-
O2	Healthy	Inakadate, Aomori, Japan	-
O3	Diseased	Kuroishi, Aomori, Japan	-
O4	Healthy	Kuroishi, Aomori, Japan	-
O5	Healthy	Kuroishi, Aomori, Japan	-
O6	Healthy	Osato, Miyagi, Japan	-
O7	Healthy	Kuroishi, Aomori, Japan	-
O8	Healthy	Osato, Miyagi, Japan	-
O9	Healthy	Toyoma, Miyagi, Japan	-
O10	Healthy	Osato, Miyagi, Japan	-
O11	Healthy	Osato, Miyagi, Japan	-
O12	Healthy	Osato, Miyagi, Japan	-
O13	Healthy	Osato, Miyagi, Japan	-
O14	Healthy	Osato, Miyagi, Japan	-
O15	Healthy	Osato, Miyagi, Japan	-
O16	Healthy	Osato, Miyagi, Japan	-
O17	Healthy	Osato, Miyagi, Japan	-
R1	Healthy	Inakadate, Aomori, Japan	-
R2	Healthy	Inakadate, Aomori, Japan	-
R3	Healthy	Inakadate, Aomori, Japan	-
R4	Healthy	Inakadate, Aomori, Japan	-
R5	Healthy	Inakadate, Aomori, Japan	-
R6	Diseased	Inakadate, Aomori, Japan	-
R7	Healthy	Kuroishi, Aomori, Japan	-
R8	Healthy	Inakadate, Aomori, Japan	-
R9	Diseased	Inakadate, Aomori, Japan	-
R10	Diseased	Kuroishi, Aomori, Japan	-
R11	Healthy	Inakadate, Aomori, Japan	-
R12	Healthy	Inakadate, Aomori, Japan	-
R13	Healthy	Inakadate, Aomori, Japan	-
R14	Healthy	Kuroishi, Aomori, Japan	-
R15	Healthy	Inakadate, Aomori, Japan	-
R16	Healthy	Inakadate, Aomori, Japan	-
R17	Healthy	Inakadate, Aomori, Japan	-
R18	Healthy	Kuroishi, Aomori, Japan	-
R19	Healthy	Inakadate, Aomori, Japan	-
W1	Healthy	Osato, Miyagi, Japan	-
W2	Healthy	Osato, Miyagi, Japan	+
W3	Healthy	Osato, Miyagi, Japan	++
W4	Diseased	Inakadate, Aomori, Japan	-
W5	Healthy	Inakadate, Aomori, Japan	++
W6	Diseased	Osato, Miyagi, Japan	-
W7	Healthy	Inakadate, Aomori, Japan	-
W8	Healthy	Osato, Miyagi, Japan	-
W9	Healthy	Osato, Miyagi, Japan	-
W10	Healthy	Toyoma, Miyagi, Japan	-
W11	Healthy	Osato, Miyagi, Japan	-
W12	Diseased	Toyoma, Miyagi, Japan	++
W13	Healthy	Osato, Miyagi, Japan	-
W14	Healthy	Osato, Miyagi, Japan	-
W15	Healthy	Osato, Miyagi, Japan	-
W16	Healthy	Osato, Miyagi, Japan	-
W17	Healthy	Osato, Miyagi, Japan	-
W18	Healthy	Osato, Miyagi, Japan	-
W19	Healthy	Osato, Miyagi, Japan	-
W20	Healthy	Inakadate, Aomori, Japan	-
W21	Healthy	Osato, Miyagi, Japan	-

(TABLE S1 cont.)

Isolate ID	Condition of source plant	Collection place	Biocontrol efficacy
W23	Healthy	Osato, Miyagi, Japan	-
W24	Healthy	Osato, Miyagi, Japan	-
W25	Healthy	Osato, Miyagi, Japan	-
W27	Healthy	Osato, Miyagi, Japan	-
W28	Healthy	Osato, Miyagi, Japan	-
W29	Healthy	Osato, Miyagi, Japan	-
W32	Healthy	Osato, Miyagi, Japan	-
W33	Healthy	Osato, Miyagi, Japan	-
W34	Healthy	Osato, Miyagi, Japan	-
Y10	Healthy	Inakadate, Aomori, Japan	-
Y11	Healthy	Kuroishi, Aomori, Japan	-

Supporting information Materials and Methods

Phylogenetic analysis

The sequence data of the intergenic spacer (IGS) region from a total of 29 taxa including outgroups (*Fusarium redolens* NRRL 31075 and *F. hostae* NRRL 29889) were used phylogenetic analyses. Except for the sequence of W5, W3, a25, a29 and Fo47, all sequence data were downloaded from NCBI database (accession number of each data is described in Fig S2). All sequences were aligned with MAFFT v7.222 using L-INS-i strategy (7), truncated at the 5'- and 3'-ends, and manually modified when necessary using MEGAX (8). For maximum likelihood (ML) and Bayesian phylogenetic analysis, general time-reversible (GTR) with gamma distribution (shape parameter=1.0193) was determined as the best-fit model by hierarchical likelihood ratio tests (hLRTs) using MrModeltest v2.4 (9) and PAUP* version 4.0a (build 168) (10). Heuristic search of maximum parsimony analysis was performed for 1000 replications with TBR branch-swapping using PAUP*, followed by bootstrap analysis to the consensus tree. Maximum likelihood analysis was performed using all-in-one option of RAxML-NG (11) with 1000 bootstrapping. Bayesian inference using Metropolis-coupled Markov chain Monte Carlo (MCMCMC) methods was performed using MrBayes v3.2.7a (12), with four Markov chains for 1×10^6 generations.

Supporting information References

1. Nirenberg HI. 1976. Untersuchungen über die morphologische und biologische Differenzierung in der *Fusarium*-Sektion *Liseola*. Mitteilungen aus der Biologischen Bundesanstalt Für Land- und Forstwirtschaft (Berlin-Dahlem) 169: 1-117.
2. Inami K, Yoshikawa-Akiyama C, Morita Y, Yamasaki M, Teraoka T, Arie T. 2012. A genetic mechanism for emergence of races in *Fusarium oxysporum* f. sp. *lycopersici*: Inactivation of avirulence gene *AVR*1 by transposon insertion. PLOS ONE 7: e44101.
3. Kashiwa T, Inami K, Fujinaga M, Ogiso H, Yoshida T, Teraoka T, Arie T. 2013. An avirulence gene homologue in the tomato wilt fungus *Fusarium oxysporum* f. sp. *lycopersici* race 1 functions as a virulence gene in the cabbage yellows fungus *F. oxysporum* f. sp. *conglutinans*. J Gen Plant Pathol 79: 412–421.
4. Watanabe S, Kumakura K, Izawa N, Nagayama K, Mitachi T, Kanamori M, Teraoka T, Arie T. 2007. Mode of action of *Trichoderma asperellum* SKT-1, a biocontrol agent against *Gibberella fujikuroi*. J Pestic Sci 32: 222–228.
5. Namiki F, Matsunaga M, Okuda M, Inoue I, Nishi K, Fujita Y, Tsuge T. 2001. Mutation of an arginine biosynthesis gene causes reduced pathogenicity in *Fusarium oxysporum* f. sp. *melonis*. Mol Plant Microbe Interact 14: 580–584.
6. Matsuo T, Hoshikawa K. 1993. Science of the rice plant. Food and Agriculture Policy Research Center, Tokyo, Japan.
7. Katoh K, Standley DM. 2013. MAFFT multiple sequence alignment software version 7: improvements in performance and usability. Mol Biol Evol 30: 772–780.
8. Kumar S, Stecher G, Li M, Knyaz C, Tamura K. 2018. MEGA X: molecular evolutionary genetics analysis across computing platforms. Mol Biol Evol 35: 1547–1549.

9. Nylander JAA. 2004. MrModeltest v2. Program distributed by the author. Evolutionary Biology Centre, Uppsala University. https://github.com/nylander/MrModeltest2

10. Swofford DL. 2002 PAUP*: phylogenetic analysis using parsimony and other 597 methods, 4.0β10. Sinauer, Sunderland

11. Kozlov AM, Darriba D, Flouri T, Morel B, Stamatakis A. 2019. RAxML-NG: a fast, scalable and user-friendly tool for maximum likelihood phylogenetic inference. Bioinformatics 35: 4453‑4455.

12. Ronquist F, Teslenko M, van der Mark P, Ayres DL, Darling A, Höhna S, Larget B, Liu L, Suchard MA, Huelsenbeck J. 2012. MrBayes 3.2: efficient Bayesian phylogenetic inference and model choice across a large model space. Syst Biol 61: 539–542.
